# Engineering Ag43 Signal Peptides with Bacterial Display and Selection

**DOI:** 10.3390/mps6010001

**Published:** 2022-12-23

**Authors:** Darius Wen-Shuo Koh, Jian-Hua Tay, Samuel Ken-En Gan

**Affiliations:** 1 Antibody & Product Development Laboratory, Agency for Science, Technology, and Research (A*STAR), Singapore 138671, Singapore; 2 James Cook University, Singapore, Singapore 387380, Singapore; 3 Zhejiang Bioinformatics International Science and Technology Cooperation Center, Wenzhou-Kean University, Wenzhou 325015, China; 4 Wenzhou Municipal Key Lab of Applied Biomedical and Biopharmaceutical Informatics, Wenzhou-Kean University, Wenzhou 325015, China

**Keywords:** single-cell screening, protein surface display, engineering signal peptides, Ag43 autotransporter, error-prone PCR

## Abstract

Protein display, secretion, and export in prokaryotes are essential for utilizing microbial systems as engineered living materials, medicines, biocatalysts, and protein factories. To select for improved signal peptides for *Escherichia coli* protein display, we utilized error-prone polymerase chain reaction (epPCR) coupled with single-cell sorting and microplate titer to generate, select, and detect improved Ag43 signal peptides. Through just three rounds of mutagenesis and selection using green fluorescence from the 56 kDa sfGFP-beta-lactamase, we isolated clones that modestly increased surface display from 1.4- to 3-fold as detected by the microplate plate-reader and native SDS-PAGE assays. To establish that the functional protein was displayed extracellularly, we trypsinized the bacterial cells to release the surface displayed proteins for analysis. This workflow demonstrated a fast and high-throughput method leveraging epPCR and single-cell sorting to augment bacterial surface display rapidly that could be applied to other bacterial proteins.

## 1. Introduction

Augmenting protein display and secretion by bacteria is desirable for many applications, such as developing whole-cell catalysts [1,2,3] in industrial biotechnology and displaying vaccine subunits [4] as living medicines. Many such approaches, from optimizing the ribosome binding site, promoter, and codon usage to screening different natural signal peptides or autotransporter β-barrel domains to optimize the autotransporter-mediated display of recombinant secreted proteins [2,5,6,7,8], demonstrate the interest in this area.

To enable the programming of cells to fabricate engineered living materials (ELMs) [9,10,11,12], deliver protein therapeutics [13,14,15,16], test mutant protein variants faster [17,18,19,20,21,22,23,24], optimize bioprocess parameters [25,26], perform biochemical reactions [1,3], and construct biosensors [7,27] require the display or secretion of proteins in both eukaryotes and prokaryotes. *Escherichia coli* (*E. coli*) remains one of the best-characterized model organisms for engineering the secretion of proteins into the extracellular environment [28]. Given that proteins can be either displayed on the surface of cells or released into the medium [29,30], there is the opportunity to harvest these proteins without a laborious lysis step that could involve contamination of endogenous intracellular proteins. With extracellular expression reported to circumvent cytosolic aggregation and proteolysis of proteins, higher titers of untruncated protein-based polymeric materials [30] could be harvested from the media.

The secretion and protein export pathways of *E. coli* are divided into Types I, II, III, IV, and V (reviewed in [28,31]) pathways. Many attempts were made to engineer these pathways for heterologous protein production and export, mainly targeting the Type I system, where attempts to modify it usually encompass the mutagenesis of secretion carrier proteins [32], leader peptides [33,34], and chaperones [35,36,37] in the Sec/Tat pathways followed by the selection and screening systems based on high throughput plate screening or antibiotic survival selection assays.

The natural Ag43 autotransporter system usually consists of a signal peptide (to mediate targeting by chaperones), an N-proximal passenger domain (α^43^), an auto chaperone domain, and a C-terminal Ag43 β-barrel domain (β^43^) [8,29,38]. It is abundantly displayed on the bacteria and belongs to the Type V subtype A secretion system, relying on the Sec chaperone system [8,31,38]. The Ag43 autotransporter enables protein secretion into the media through a proteolytic site in α^43^ [8,29,38], where the absence of this domain would allow for heterologous proteins to be more reliably displayed [8,29]. One advantage of the Ag43 system is its ability to display a vast repertoire of disulfide-containing proteins as determined by single-cell flow cytometry [8,39]. With the many uses of the Ag43 autotransporter system, such as engineering the specific gut colonization of probiotic *E. coli* Nissle 1917 strain to display adhesive proteins to alleviate dextran sulfate sodium-induced colitis in murine models [16], and thermostable β-glucosidase display for whole-cell biocatalyst transformation of cellulose into simple sugars [1], there remain many applications. Examples in the heterologous expression of proteins are that they could also be toggled between display and secretion modes with conditional expression of tobacco etch viral protein proteases [29] and as ELMs in allowing for microbial optogenetic lithography of complex spatial biofilm patterns [11].

One advantage of engineering the Ag43 system is that the protein of interest can be physically linked to the cell, thus allowing for fluorescence-activated cell sorting (FACs)—a powerful multiparametric and (semi) quantitative tool [40,41] widely used in bio-foundries [42,43] to develop microbes resistant to low pHs [43], superfast enzymes [44], epigenetic probes [45], ribosensors [46], and LacI [47] with expanded ligand responsiveness which could not otherwise exist in nature.

To engineer the Ag43 system for a more productive display and export of a moderately sized passenger protein or sfGFP-beta-lactamase fusion (56 kDa) beyond the cell wall, we utilized error-prone polymerase chain reaction (epPCR) with FACs in a proof-of-concept experiment for the selection of augmenting protein export in *E. coli* with using plate reader high-throughput measurements. Although the switching of natural signal peptides to optimize signal peptides of autotransporters was previously made [2,8], the methodology using epPCR to mutate signal peptides of autotransporters with single-cell selections reported here is not previously reported to our knowledge.

## 2. Materials and Methods

### 2.1. Molecular Cloning

**Figure 1:**
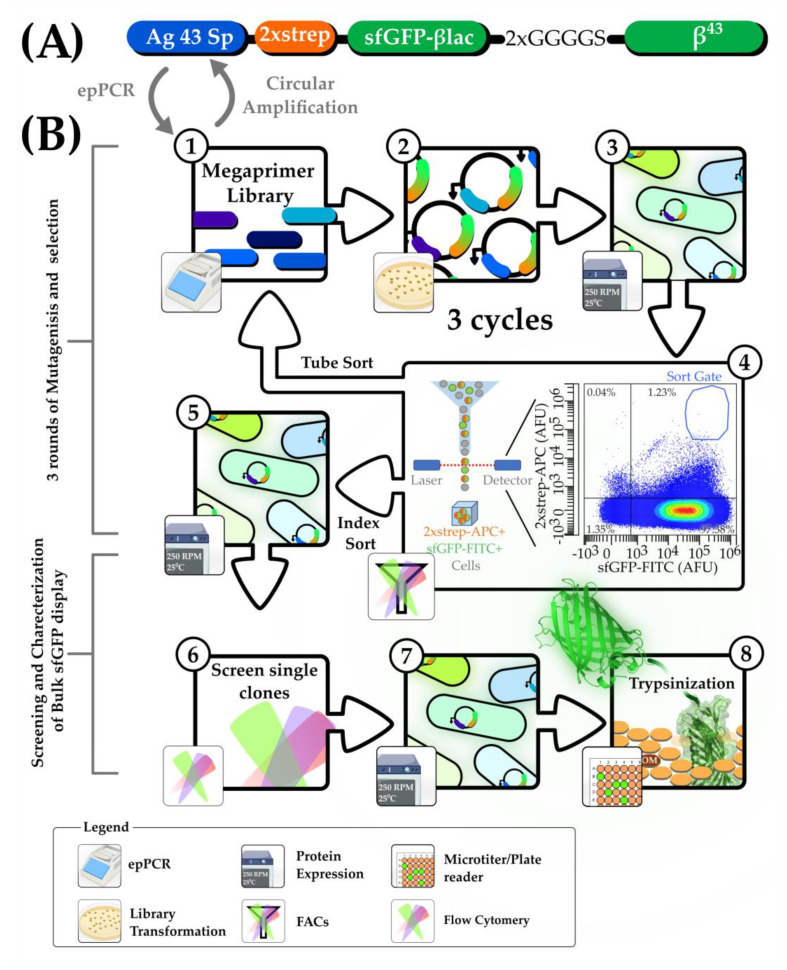
Schematic of the workflow to generate and select Ag43 signal peptide (Ag43 Sp) mutant sequences for augmented display. (**A**) Illustration of the critical features in the Ag43 autotransporter system: Ag43 signal peptide (Ag43 Sp), twin-strep tag-II (2×strep), sfGFP, β-lactamase (βlac), 2× GGGGS linker, and β-barrel domain(β^43^), which also tethered the protein onto the outer membrane. (**B**) The workflow to generate the Ag43 signal peptide mutations in this study; steps are labelled in circular labels. (**Step 1–4 mutagenesis and selection, repeated for three cycles**). Step 1, megaprimer libraries were generated with error-prone PCR (epPCR) to create megaprimers used for circular amplification (using wild-type plasmid pCDF_Ag43sp_2×strepSfGFPβLac as template) to generate mutant signal peptides. Steps 2 and 3, transformation and harvesting of mutant colonies for subsequent expression with 0.5 mM IPTG at 25 °C, 250 rpm for 5 to 6 h. In step 4, mutant Ag43 bacteria libraries were sorted with FACs. (**Steps 5–8, screening and characterization of isolated index sorted clones**) Steps 5–6, 43 clones (index sorted into microwell plates) were expressed and screened with FACs. In steps 7–8, three of the best clones (with the best flow-cytometric profiles) were selected and expressed in 50 mL falcon tubes and trypsinized to release displayed sfGFP on the cell surface. Trypsinized cell supernatants were scanned for sfGFP signals with a plate reader and subjected to native SDS-PAGE for analysis.

To design the Ag43 autotransporter display system, a codon-optimized super-folding green fluorescent protein (sfGFP) sequence was flanked by the native nucleotide sequence (NCBI GeneID: 94650) for the N-terminal Ag43 signal peptide, twin strep-tag II (2×strep), and C terminal β^43^ domain as previously performed [8] (see Figure 1A under the Results Section). The designed construct was gene-synthesized and cloned into the pCDF plasmid backbone using the Ncol and XhoI restriction sites added by Genscript with an alanine codon added after the first methionine in the signal peptide to ensure in-frame with the Ncol restriction site.

To create the final construct used in this study (pCDF_Ag43sp_2×strepSfGFPβLac, Figure 1A), the β-Lactamase gene was fused to the 3′ end of the sfGFP protein to make a moderately sized 56 kDa model passenger protein by first mutating pCDF_Ag43sp_2×strepSfGFPβLac by primers: Ag43LinkBlac_F + Ag43LinkBlac_R (primer sequences are listed in Table S1) using the Agilent QuikChange SDM kit following the manufacturer’s instructions (Agilent, Singapore, Singapore; Cat: STR_210519). This was followed by amplifying the backbone plasmid and β-Lactamase gene (amplified from Addgene plasmid RRID: #52656) with primer set Ag43BBGib_F+Ag43BBGib_R and Ag43BlacGib_F + Ag43BlacGib_R, respectively (primer sequences and corresponding PCR profiles are listed in Table S1) using the Platinum SuperFi II Green PCR master mix (ThermoFisher, Singapore; Cat: 12369010). The gel-extracted plasmid backbone and insert were assembled with 2× NEB HiFi DNA assembly kit following the manufacturer’s instructions.

Plasmids were prepped as previously described [48] using *E. coli* BL21 (DE3) previously described in [49]. DNA quantifications were performed with the Qubit DS HS or BR DNA kit (Invitrogen^TM^, Singapore) following the manufacturer’s instructions. High throughput optical density (OD) normalization steps were performed with the OT2 (OpenTrons, Long Island City, NY, USA) liquid handling automation system using custom python scripts. For pCDF-based plasmids, 200 μg/mL of spectinomycin was used. All centrifugation procedures were performed at 8 k rpm for 5 min or 4 k rpm for 1 h in 1.5 mL microfuge tubes or 2 mL Deepwell (NEST, Cat: 503501) plates for low- and high-throughput procedures, respectively.

### 2.2. Mutant Library Construction

Plasmids isolated from the gene-synthesized clones and FACS-sorted *E. coli* BL21 (DE3) cells were subjected to high-fidelity Q5 Polymerase (NEB, Singapore; Cat: M0491L) PCR to generate 269 bp templates of the Ag43 signal peptide (Figure 1A). To generate the mutant Ag43 signal peptide library, 2.5 ng of the template per 100 µL were then subjected to epPCR (with primer set Ag43epPCR_F+Ag43epPCR_R using the GeneMorph II epPCR kit (Agilent, Singapore, Cat: STR_200550). The epPCR profile and reaction is further described in Table 1 below.

Amplified mutant Ag43 signal peptide gene libraries were analysed using gel electrophoresis and GelApp [50] prior to gel extraction. Error rates were estimated by calculating the PCR duplications required to achieve the final gel extracted products yield from 2.5 ng of template, according to the GeneMorph II epPCR kit manufacturer’s instructions. The mutant Ag43 signal peptide libraries were used as megaprimers for the circular PCR amplification using the wild-type pCDF_Ag43sp_2×strepSfGFPβLac as a template with the Platinum SuperFi II Green PCR master mix. The reaction mix and PCR conditions are detailed in Table 2. Mutant plasmid libraries were then subjected to a PCR clean-up, eluted with 40 μL nuclease-free water, and treated with 20 U of DpnI (NEB, Singapore; Cat: R0176L) for 30 min according to the manufacturer’s instructions.

### 2.3. Expression of Autotransporter System

To generate mutant clones for expression and selection from FACs, ~100 ng of Ag43 signal peptide mega primer libraries PCR amplified plasmids were transformed into 100 μL of competent BL21 (DE3) cells [49].

Transformed cells were then divided and plated onto three plates (Biomedia, Singapore; 100 × 15 mm; Cat: BMH.990000PQ-20) of lysogeny broth (LB) agar containing the appropriate antibiotic and incubated at 37 °C overnight. Colonies were manually counted and evaluated with the APD Colony Counter App [51]. Transformed cells were collected each round and pooled into LB containing 200 μg/mL spectinomycin. To preserve the diversity of the mutant libraries, induction was performed immediately by diluting the pooled cultures to OD_600_ 0.4 in 5 mL of LB containing 0.5 mM of Isopropyl β-D-1-thiogalactopyranoside (IPTG) and appropriate antibiotics to be incubated at 25 °C, 250 rpm (in a Benchtop ES-20, Orbital Shaker-Incubator, Latvia) for 5 to 6 h. The controls: untransformed (no Plasmid or NoP) and wild-type plasmid containing BL21 (DE3) were induced in the same manner with no antibiotics included for the untransformed cells.

### 2.4. Cell Sorting and Flow Cytometry

For flow cytometric assessments of the heterologous display of sfGFP on mutant clones, 500 μL of cells were first normalized to OD_600_ 0.4. Next, cells were washed twofold with 500 μL of a phosphate-buffered solution (PBS) containing protease inhibitor (PI). Next, staining was performed with 1.5 μg/mL of streptavidin conjugated to Alexa Fluor^TM^ 647 (Invitrogen^TM^, Singapore) for an hour, followed by two additional washes with PI containing PBS before acquisition and analysis on the CytoFlex Srt (Beckman Coulter, Singapore; Model: V5-B2-Y5-R3). Stained cells were then stored at 4 °C for up to 24 h. Cell sorting was performed at OD_600_ 0.2.

### 2.5. Flow Cytometric and FACs Assay Setup

Streptavidin-bound cells were detected in APC (allophycocyanin channel, 660/20 bandpass filter), herein called 2×strep-APC, and sfGFP displayed on the cell surface was detected in FITC (fluorescein channel, 525/40 bandpass filter), herein called sfGFP-FITC, while the control BL21 (DE3) was set for baseline adjustment. After 5 million events were collected, dual color flow cytometric density plots were generated. Next 2×strep-APC+ and sfGFP-FITC+ cells were sorted for at least an hour (threshold for sort-gate: 2×strep-APC ~3 × 10^4^ to 5 × 10^5^, sfGFP-FITC intensity ~4 × 10^4^ to 3 × 10^6^) in purity mode into FACs tubes between 3 rounds of mutagenesis and FACs selection of Ag43 signal peptide mutant libraries. In the first two rounds, cells were tube-sorted. Next, cells were mixed with 20 mL of media between two 50 mL Falcon™ tubes (Corning, USA). In the last round, cells from round 2 were induced the following day and FACs-enriched for 2×strep-APC+ and sfGFP-FITC+ clones. Clones were then miniprepped, subjected to a round of mutagenesis, allowed to express, and index-sorted into 96-well microtiter plates at the end of the third round of sorting. Index-sorted cells were transferred to deep-well plates containing 400 μL of media. Cells in either tubes or deep-well plates were incubated at 37 °C, 250 rpm overnight in the ES-20, Orbital Shaker-Incubator and MaxQ 8000 incubator (ThermoFisher, USA), respectively.

### 2.6. Screening of the Mutant Clones

For all experiments involving the expression of clones for screening and characterization, the MaxQ 8000 incubator was used. Overnight cultures of 43 mutant clones (22.4% of positive viable clones were recovered) isolated with the CytoFlex Index sorting function were inoculated as 1% starter culture in 2 mL LB medium (in 14 mL flacon snap cap tubes; Corning, USA) induced between OD_600_ 0.6 and 0.8. Cells were induced with 0.5 mM IPTG at 25 °C, 250 rpm for 5 to 6 h.

To characterize the total functional sfGFP displayed, 1% starter cultures were inoculated in 10 mL of LB (in 50 mL falcon tubes; Corning, USA) and induced similarly as were performed for initial screens. After 6 and 12 h of induction, 5 mL of culture was collected at the respective time points. Trypsinization was previously performed and found suitable to characterize autotransporter-based systems in *E. coli* [52,53]. To determine total fluorescent sfGFP on the surface of cells, the bacteria cells were normalized by OD where they were first spun down and resuspended to the equivalent of OD 4 or OD 8 with 400 μL of 2.5 g/L of Trypsin solution (Nacali Tesque, Japan; Cat: 32777-44, Lot No. L0F24273), followed by incubation at 37 °C, 250 rpm for 2 h. The supernatant of trypsinized cells sfGFP was separated by centrifugation while the cell pellet was resuspended with the same volume of fresh trypsin solution.

For cells trypsinized at OD 4, 150 μL of trypsinized cell supernatant or trypsinized cell pellet resuspended in fresh trypsin solution were analysed for sfGFP fluorescence (485/20 nm, emission 528/20 nm; optimized gain manually set at 75) with a TECAN SAFIRE II plate-reader (TECAN, Switzerland). Cleaved sfGFP from the supernatant of cells trypsinized at OD 8 (gave bright bands from initial optimizations using bacteria cells, including the wild-type signal peptide) were used for SDS-PAGE separation of functional sfGFP. The cleaved sfGFP-containing liquid was mixed with 5× native loading dye (Tris-HCl 250 mM, pH 6.8; 30%, glycerol; 0.02% Bromophenol blue) without a boiling step and loaded (13 μL) onto precast 12% SDS-PAGE gels (BioRad, Singapore; Cat: 4561046). Gels were illuminated with a blue light transilluminator. ImageLab^TM^ (BioRad, USA) and GelApp [50] were used for densitometric analysis and band analysis, respectively.

To sequence the mutants, Sanger sequencing (1st Base, Singapore) was performed using the Platinum SuperFi II Green PCR master mix (following the manufacturer’s protocol, using primer set: Ag43SpSeq_F+Ag43SpSeq_R; primer sequences and corresponding PCR profiles are listed in Table S1) since the quality of BL21 (DE3) isolated plasmids was poor. Sequences were analysed with a locally installed version of YAQAAT [54] (using the wild-type signal peptide sequence as a template) and DNAApp [55] for analysis.

### 2.7. Data Analysis

The plate-reader and densitometric data were analysed using GraphPad Prism Version 9.0.0; a one-way analysis of variance (ANOVA) with Dunnett’s comparison test against the induced wild type was performed. FACS and flow-cytometric plots were generated with the CytExpert SRT version 1.0.2 and FlowJo^TM^ version 10.8.1 software.

## 3. Results and Discussion

### 3.1. FACs Selection for Single-Cell Mutants with High Levels of Display and Expression

The native *E. coli* Ag43 autotransporter displays heterologous proteins on the bacteria cell surface and is a good marker for our FACs-sorted high throughput selection of single-cells. To detect extracellular display, fluorescent-tagged streptavidin and 2×strep-tag fused to a reporter of total functional protein expression sfGFP were used (an overall flow of the methodology is shown in Figure 1). Three rounds of selection (R1, 2, and 3) of reiterative mutagenesis and selection for single-cell mutants with extreme upper right quadrant sfGFP-FITC and 2×strep-APC signals screening of 2900–8700 colonies/round showed evident enrichment. The average error rates of mutant libraries were estimated to be between medium to high (4.5–16 average errors/1000 bp) range across all three rounds of mutagenesis.

**Figure 2:**
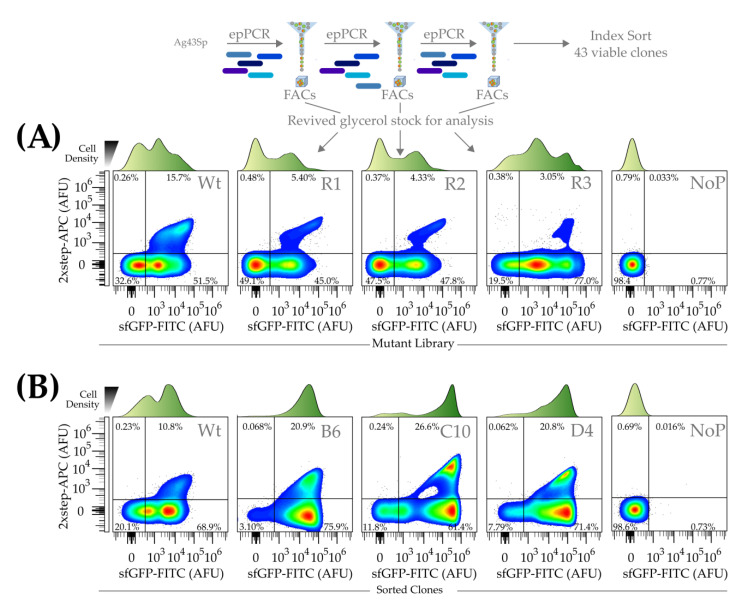
**Representative flow cytometric charts of mutant Ag43 signal peptide BL21 (DE3) libraries.** (**A**) Flow cytometry of sorted mutant Ag43 signal peptide libraries. R1, R2, and R3 corresponded to cell populations with mutant libraries that underwent 1, 2, and 3 rounds of epPCR mutagenesis and FACs selection. “Wt” and “NoP” corresponded to populations containing wild-type Ag43 signal peptide and no plasmid, respectively. The population of single-cells 2×strep-APC+/sfGFP-FITC+ (corresponded to higher fluorescent streptavidin binding and sfGFP fluorescence) and those exhibited extreme sfGFP-FITC signals were found to reduce and increase, respectively, over the three rounds of selection. To express the displayed proteins, 100 μL of frozen bacteria glycerol stocks were used as a starter culture to inoculate 10 mL cultures in Falcon tubes. (**B**) Flow cytometry of the top three mutants (B6, C10, and D4) out of 43 screened clones with the AFU, arbitrary fluorescence units. Histograms of the density of cells expressing different levels of sfGFP-FITC fluorescence are shown above the respective plots (Figure 2 is representative of triplicates).

To compare the selected clones of the various rounds, they were glycerol stocked, thawed, expressed, and subjected to flow cytometry (Figure 2A). We noticed 2×strep-APC+/sfGFP-FITC+ wild-type cells to exhibit a wave-tide-like topology (Figure 2A) around 1 × 10^4^ arbitrary fluorescent units (AFUs) on the flow cytometry plots.

The mutant 2×strep-APC+/sfGFP-FITC+ and 2×strep-APC-/sfGFP-FITC+ sub-populations progressively increased in sfGFP-FITC signal strength over the three rounds of mutagenesis and selection (Figure 2A). Single cells within R1 and R2 mutant pools positive for 2×strep-APC+ formed a sizable population with fluorescence ~1 × 10^5^ AFUs compared to ~1 × 10^4^ AFUs in the wild-type, demonstrating the successful selection pressure applied for cells with stronger sfGFP signals (Figure 2A). Between R1 and R2, the increased single-cell sfGFP-FITC fluorescence was accompanied by a corresponding decrease in 2×strep-APC+/sfGFP-FITC+ single-cell populations. This could be due to the number of cells expressing 2×strep-APC signals for surface display being decreased (Figure 2A) or fluorescence quenching. This can be observed with the boomerang-shaped dot plots of the 2×strep-APC+/sfGFP-FITC+.

By the third round (R3), there was a significant decrease of 2×strep-APC+/sfGFP-FITC+ populations accompanied by major populations that were either 2×strep-APC+ or 2×strep-APC- to express sfGFP-FITC around 1 × 10^5^ AFUs which was 2–10-fold higher than the majority in the wild-type control. Given the inverse relationship between 2×strep-APC+ and sfGFP-FITC+ signals, there was an unexplained tradeoff in the selection.

In the final third round of selection, we isolated 43 viable clones sorted into 96-well plates (Figure 2B and  Figures S1–S3), identifying three representative mutant clones: B6, C10, and D4, which displayed sfGFP-FITC+/2×strep-APC+ population enrichments of 20.9%, 26.6%, and 20.8% the initial screens, respectively (Figure 2B). Within the population of sfGFP-FITC+/2×strep-APC+ and sfGFP-FITC+/2×strep-APC-, a sizable population of B6, C10, and D4 cells displayed sfGFP-FITC fluorescence around 3 × 10^4^, 2 × 10^5^, and 1 × 10^5^ AFUs, respectively. In contrast, most of the cells in the wild-type population were around 1 × 10^4^ AFUs for sfGFP-FITC fluorescence.

### 3.2. Characterization of Bulk sfGFP Expression in Mutants

**Figure 3:**
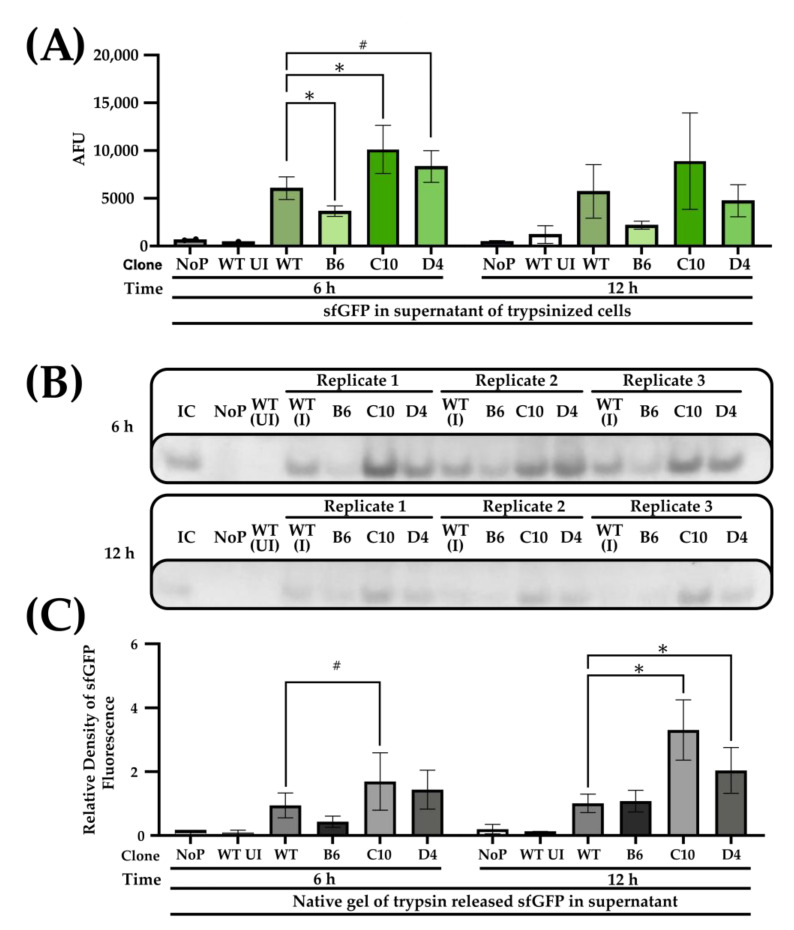
**Quantification and SDS PAGE of trypsinized bacterial cells to trypsinized sfGFP in the supernatant.** (**A**) Mean total sfGFP fluorescence of cell supernatants after trypsinization. Bacterial cells of the wild-type (Wt, induced) Ag43 signal peptide was compared with mutant (B6, C10, and D4) Ag43 variants. Uninduced wild-type Ag43 signal peptide (Wt UI) and BL21 (DE3) untransformed or with no plasmid (NoP) served as negative controls. (**B**) Native SDS-PAGE gel of trypsinized bacterial cell supernatants. sfGFP were excited by the blue light. A common internal control (IC) corresponding to Replicate 1 of WT (I) was included to facilitate comparison between gels. (**C**) Densitometric analysis of sfGFP signals is shown in (**B**). Except for Wt UI and NoP, WT and mutant variants were performed in triplicates and sextuplicate, respectively. One-way ANOVA and Dunnett post hoc test were used for comparing the wild-type and mutant variants (# < 0.1, * *p* < 0.05).

As single-cell population data may not translate to bulk titers of functional displayed sfGFP and to further confirm that sfGFP were indeed displayed on the surface, we trypsinized untransformed BL21 (DE3), wild-type (induced and uninduced), and mutant clones (B4, C10, and D4) with Ag43 leader signal peptide constructs that were induced for 6 and 12 h, respectively. Since sfGFP is resistant to trypsinization [56], it would remain intact and be released while the bacterial cell walls remain intact. We could verify that the protein was released from the bacterial surface by analyzing the supernatant with a plate reader and native SDS-PAGE (boiling omitted to preserve sfGFP functionality). To quantify the density of bands in native SDS-PAGE across different gels, the first induced wild-type replicate (Wt I-R1) was used as an internal control for normalization during densitometric analysis (Figure 3B).

Examining trypsinized bacteria cell supernatants at the 6- and 12-h timepoints by plate-reader microtiter assays showed that at the 6-h time point, sfGFP fluorescence of C10 and D4 were significantly higher by 1.7 (*p* < 0.05), and 1.4 (*p* < 0.1) folds than the induced wild-type controls (Wt). On the contrary, B6 had lower fluorescence than the wild-type, with similar trends observed for the 12-h timepoint experiments for all the three mutants B6, C10, and D4 but with readings of 0.4 and 1.6 and 0.8 folds to the wild-type, and without statistical significance. Considering that the readings from the supernatant also showed higher cleaved sfGFP levels, the proteins were reasonable assumed to be displayed on the cell surface.

**Figure 4:**
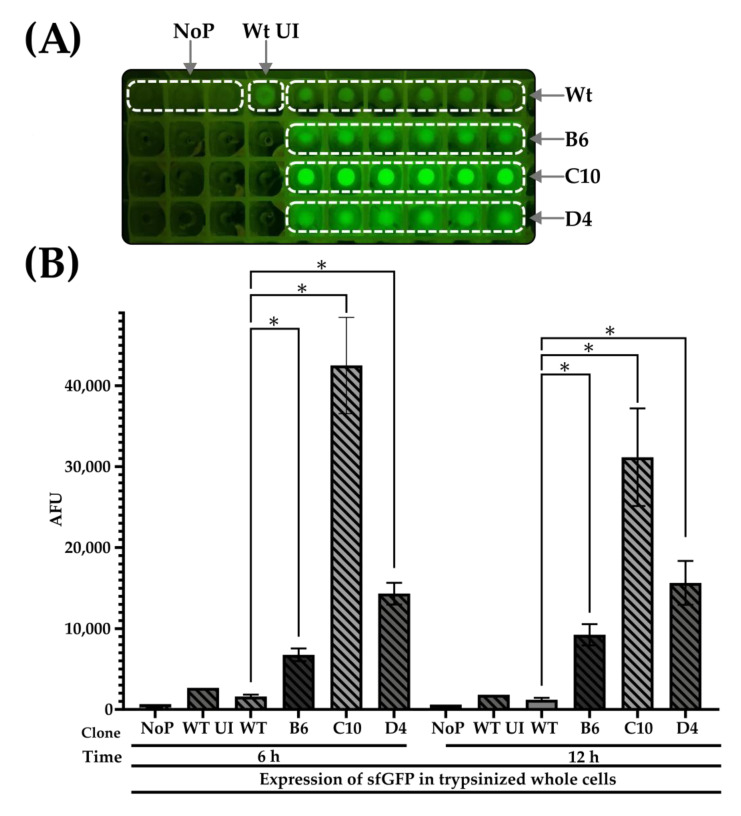
**Quantification of whole bacterial cell sfGFP.** (**A**) A photograph of pelleted trypsinized whole bacterial cells (from Figure 3) expressing sfGFP fused to either wild-type (Wt, induced) or mutant (B6, C10, and D4) Ag43 sp, Pelleted cells with uninduced SfGFP fused to wild-type Ag43 Sp (Wt UI) or BL21 (DE3) without plasmid (NoP) served as negative controls. (**B**) Intracellular sfGFP signals from pelleted whole cells (from A, resuspended in trypsin solution) were quantified with a microplate reader. Except for Wt UI and NoP, WT and mutant variants were performed in triplicates and sextuplicates, respectively. One-way ANOVA and Dunnett post hoc test were used to compare the wild-type and selected mutant variants (* *p* < 0.05). Analysis of the sequences showed Clone C10 to have five missense nucleotide mutations 70 G > A, 74 T > G, 86 G > C, 104 G > A, 116 T > A, and 121 C > G leading to amino acid substitutions of A24T, S25A, R29P, G35D, V39D, and L41V (Figure 5). Three other clones were sequenced and found to harbor the same mutations as C10: B2, B11, and C2 (Table 3, Figures S1 and S2). On the other hand, Clone D4 had just one silent mutation, G156C (Figure 5), and carried the same mutation as Clone B9 (Table 3, Figure S2). The 156 G > C mutation led to a change from a high (CTG) to low-usage codon (CTC) for *E. coli* (using strain *E. coli O157:H7 str. EDL933* as a reference) with frequencies of 51.1% and 10.5%, respectively [59,60]. The presence of silent mutations leading to codons with lower usages was previously reported to increase the ability of protein secretion tags to export proteins extracellularly by 1.6 folds [32]. It was recently reported to affect mutational fitness in eukaryotes [61] or in some studied cases, the folding of microbial proteins [62].

Observing trypsinized cells to glow intensely under blue light and populations of 2×strep-APC- cells to be sfGFP-FITC+, we also sought to quantify the intracellular fluorescence of post-trypsinized whole cells (with a plate reader, Figure 4A). Intracellular fluorescence of all mutants was significantly higher than the wild-type (*p* < 0.05) (Figure 4B), with B6, C10, and D4 clones exhibiting 4.2, 26.6, and 8.9 folds than the wild-type at the 6 h timepoint. At 12 h, point B6, C10, and D4 clones showed 7.7-, 25.8-, and 13-fold more fluorescence than the wild-type. The data suggest that the mutant Ag43 signal peptides were more efficient than the wild-type after the three rounds of selections. Intriguingly, the observed boosts in intracellular sfGFP accumulation were asymmetric relative to the modest improvements in the display of the sfGFP–beta–lactamase fusion. This hinted at the greater complexity of signal peptide-directed protein export pathways, particularly for the fast-folding sfGFP in *E. coli* [57,58].

The results from the Native SDS-PAGE were congruent with the trends found in the plate-reader-based microtiter assay. At the 6-h time point, densities of the fluorescent signal generated by sfGFP and separated by native SDS-PAGE showed B6, C10, and D4 to be 0.5-, 1.8-fold, and 1.5-fold higher than the wild-type (Figure 3C) with only the change in C10 to be significant (*p* < 0.1). At the 12-h time, sfGFP fluorescence densities of C10 and D4 were significantly increased by 3.2- and 2-fold. B6 was, however, unchanged compared to Wt (Figure 3B, *p* < 0.05).

**Figure 5:**
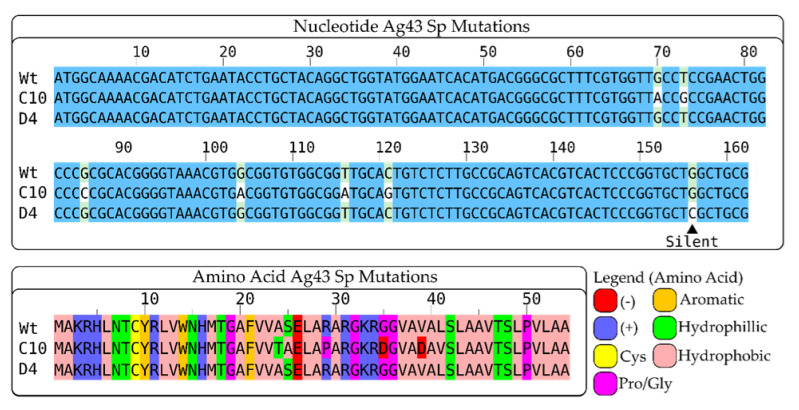
Multiple sequence alignment of the nucleotide and amino acid sequence of the Ag43 signal peptides of Clone C10 and D4. ▲ indicates the position of a silent mutation (Clone D4, G156C). Aligned amino acid sequences are colored according to the Zappo scheme. The figure was prepared with Jalview [63].

A total of 29 mutant clones were successfully sequenced (Figure 5 and Figure S4 and Table 3); Clone B6 could not be successfully sequenced while Clones B5 and D5 had wild-type signal peptide sequences. Four other clones were found to converge on the sequences of both C10 and D4 (Figure 5), which consistently gave higher readings in both whole cells and supernatants.

**Table 3:** Mutations in sequenced mutant clones and underlined clones indicate mutant clones that shared the same signal peptide mutations as clones C10 and D4.

Clone	Mutation
C10, B2, C2, and B11	70 G > A (A24T),
	74 T > G (S25A),
	86 G > C (R29P)
	104 G > A (G35D)
	116 T > A (V39D)
	121 C > G (L41V)
D4, B9	G156C (silent)

Using FACs for single-cell screening has been widely cited as a foundational tool in biofoundries by biological engineers [40,43] for the high-throughput selection of molecules with novel properties and functions. In this study, we were able to mutate and select signal peptides of autotransporters by relying on two parameters, the expression of sfGFP measured by 2×strep-APC and total sfGFP (detected as sfGFP-FITC) to generate mutations that facilitated the ability of 2×strep-APC+ single-cells to exhibit progressively greater sfGFP-FITC fluorescence over three rounds of epPCR mutagenesis and FACs screenings. In this proof-of-concept methodology, we demonstrated our workflow to generate Clones C10 and D4, which harbored five missense and one silent mutation, respectively, for moderately augmented display of the fast-folding sfGFP-beta-lactamase fusion as measured by normalized plate-reader-based analysis and native SDS-PAGE of trypsinized surface proteins. Meanwhile, we could only screen upwards of 10^5^ bacteria clones over three rounds of selection. This could be scaled up quickly as it is only limited by the plating and transformation steps and the throughput of the FACs machine, which can record around a million events in less than two minutes.

While our system was able to increase the display of sfGFP, an inordinate improvement in intracellular expression of the mutants were also observed. This suggests that our workflow could be further characterized and optimized. Further work could include the tuning and use of titrable promoters to enable more homogenous expression patterns and behavior [64] at the single-cell level. Such a scheme would also reduce the toxicity of protein expression and mitigate effects associated with heterogenous expression patterns and improve the display of the POI [2]. Another area of future work could be the degree of functional inactivation of sfGFP, which was suggested to underlie discrepancies between activity and total displayed (as measured by flow cytometry) proteins [2]. Taken together, further characterization of different POIs classes (such as different fold classes and less fast folding proteins [65,66]) to our workflow could shed insights into POIs that could benefit from our workflow.

Some methods of optimizing *E. coli* display rely on switching different autotransporters, signal peptides, or promoters [2,8]. In such studies, improvements of ~1.6-fold were reported between seven tested autotransporters. When the native signal peptide of the AIDA autotransporter was switched with the Hbp signal peptide, reduced activity was seen in the POI [2]. Similar work replacing OmpT or PelB signal peptides with the native one on Ag43 showed worse or marginally better display (0.6- and 1.10-fold display vs. the native Ag43 signal peptide) [8] were reported. Here, we demonstrated the possibility of utilizing epPCR coupled with single-cell screening to improve the display of functional sfGFP by 1.4- to 3-fold, showing that mutagenesis of existing signal peptides may be one avenue for improving display [2]. This is also independent of the natural repertoire of signal peptides, thus expanding the limited substrate breath of autotransporter-based systems [67].

In the engineering of protein secretion (in *E. coli*) into the media where screening of mutants is usually performed with selection assays utilizing solid media or microtiter plates, epPCR, or screening of single-gene knockout mutants towards improving OsmY and YebF mediated secretion [32,37] have yielded success of similar magnitudes (1.6- to 4-fold improvements in activity of secreted proteins). Such results and the simultaneous inordinate improvement in whole/intracellular sfGFP expression (up to 25-fold improvements) validates our attempt here to an extent, and also points to the challenges in engineering export systems that are complicated by the presence of extraneous domains [2,8], fast-folding folds [65,66], or inadequate recruitment/orchestration of the chaperone machinery [2,35,65], among other host factors [37]. Nonetheless, engineering the signal peptide for improved activity in the cell is possible [58] and with extensive calibration of high-throughput single-cell screening methods to correlate activity/titer [68,69], functional proteins can be displayed in much greater numbers on *E. coli*.

In conclusion, our work here serves as a nascent template towards a highly automated and scalable [43,70,71] methodology to the facile engineering of ELMs, whole-cell catalysts, and living medicines (by optimizing the display of enzymes, binders, and functional biologics). We showed here the possibility that single-cell bacterial cell sorting coupled with epPCR and the analysis of trypsinized supernatants could select for Ag43 signal peptides to increase bacterial display.

## Figures and Tables

**Table 1:** epPCR reaction and profiles for generation of mutagenic megaprimer libraries with the GeneMorph II epPCR kit.

Component	Stock Concentration	Amount
10× Mutazyme II reaction buffer	10×	10
dNTP	40 mM	2 µL
Ag43epPCR_F(5′- CTG TAG AAA TAA TTT TGT TTA ACT TTA ATA AGG AGA TAT ACC ATG -3′)	10 µm	5 µL
Ag43epPCR_R (5′- CTT TTT CAA ATT GGG GGT GTG ACC ATG CAG CGC TAG CCG CAG C -3′)	10 µm	5 µL
Mutazyme	2.5 U/μL	2 µL
Template	Variable	2.5 ng
Water	Up to 100 µL	
	**PCR Profiles**	
**Step**	**Temperature**	**Time**
Initial Denaturation		120 s
Stage 1 (Touchdown PCR, 15 cycles)		
Denaturation	95 °C	30 s
Annealing	72 °C (−1 °C each cycle)	30 s
Extension	72 °C	30 s
Stage 2 (Conventional PCR, 25 cycles)		
Denaturation	95 °C	30 s
Annealing	57 °C	30 s
Extension (269bp product)	72 °C	30 s

**Table 2:** Circular PCR reaction and profiles for generation of plasmids containing mutant Ag43 signal peptides.

Component	Stock Concentration	Amount
SuperFi II Green PCR master mix	2×	25
Mutagenized Ag43 megaprimer libraries	250 ng	Variable
Template	50 ng	Variable
Nuclease-free water	Up to 50 µL	
	**PCR Profiles**	
**Step**	**Temperature**	**Time**
Initial Denaturation	98 °C	120 s
25 cycles		
Denaturation	98 °C	30 s
Annealing	60 °C	30 s
Extension (6375 bp product)	72 °C	210 s

## Data Availability

The data for this study is available upon reasonable request to the corresponding author.

## Supplementary Materials

The following supporting information can be downloaded at: https://www.mdpi.com/article/10.3390/mps6010001/s1, Table S1 Primers and their corresponding PCR profiles, Figures S1–S3: Representative flow cytometric analysis of screened clones and Figure S4 Multiple sequence alignment of the nucleotide and amino acid Ag43 signal peptide sequences of 26 clones.
