# Supplementary material for: Engineering Ag43 Signal Peptides with Bacterial Display and Selection
Source: Methods Protoc. 2022 Dec 23;6(1):1. doi: 10.3390/mps6010001 (PMC9844295; doi:10.3390/mps6010001)
Supplement: Supplementary file 1 [file mps-06-00001-s001.zip › mps-2045305-supplementary.pdf]

Supplementary Materials

Table S1. Primers and their corresponding PCR profiles.

Ag43LinkBlac_F and Ag43LinkBlac_R	
**Component**	**Sequence**
Ag43LinkBlac_F	5’- CAG ATC CTC GCT TGC AGC AGC CCA ATG CTT AAT CAG TGA GGC ACC -3’
Ag43LinkBlac_R	5’- TGG TAT GGA CGA ATT ATA TAA AGG TAC CGG CGG AGG CTC CCA CCC AGA AAC GCT GGT GAA AGT AAA AGA T -3’

Ag43BlacGib_F and Ag43BlacGib_R	
**Component**	**Sequence**
Ag43BlacGib_F	5’- CAG ATC CTC GCT TGC AGC AGC CCA ATG CTT AAT CAG TGA GGC ACC -3’
Ag43BlacGib_R	5’- TGG TAT GGA CGA ATT ATA TAA AGG TAC CGG CGG AGG CTC CCA CCC AGA AAC GCT GGT GAA AGT AAA AGA T -3’

	**PCR Profiles**	
**Step**	**Temperature**	**Time**
Initial Denaturation		120 seconds
Stage 1(Touchdown PCR, 15 cycles)		
Denaturation	95°C	30 seconds
Annealing	72°C (-1°C each cycle)	30 seconds
Extension (850 bp Product)	72°C	25 seconds
Stage 2(Conventional PCR, 25 cycles)		
Denaturation	95°C	30 seconds
Annealing	60°C (-1°C each cycle)	30 seconds
Extension (850 bp Product)	72°C	25 seconds

Ag43BBGib_F and Ag43BBGib_R		
**Component**	**Sequence**	**Amount**
Ag43BBGib_F	5’- GGT ACC TTT ATA TAA TTC GTC CAT ACC ATG CGT -3’	
Ag43BBGib_R	5’- GCT GCT GCA AGC GAG GAT CTG TAC TTT CAG AGC GG -’3	

	**PCR Profiles**	
**Step**	**Temperature**	**Time**
Initial Denaturation		120 seconds
Stage 1(Touchdown PCR, 15 cycles)		
Denaturation	95°C	30 seconds
Annealing	72°C (-1°C each cycle)	30 seconds
Extension (5574 bp Product)	72°C	180 seconds
Stage 2(Conventional PCR, 25 cycles)		
Denaturation	95°C	30 seconds
Annealing	60°C (-1°C each cycle)	30 seconds
Extension (5574 bp Product)	72°C	180 seconds

Ag43SpSeq_F and Ag43SpSeq_R		
**Component**	**Sequence**	**Amount**
Ag43SpSeq_F	5′- ATC GAT GTC TCG ATC ACG TCG CGG GAA TTG TGA GCG GAT AAC AAT -3′;	
Ag43SpSeq_R	5′- GCA CCA GAC GGT TGC CAC AGG CAT CTT TGC TCA GCA CGC TTT GGG -3′	

	**PCR Profiles**	
**Step**	**Temperature**	**Time**
Initial Denaturation		120 seconds
Stage 1(Touchdown PCR, 15 cycles)		
Denaturation	95°C	30 seconds
Annealing	72°C (-1°C each cycle)	30 seconds
Extension (1011 bp Product)	72°C	45 seconds
Stage 2(Conventional PCR, 20 cycles)		
Denaturation	95°C	30 seconds
Annealing	60°C	30 seconds

Extension(1011 bp Product)	72°C	45 seconds

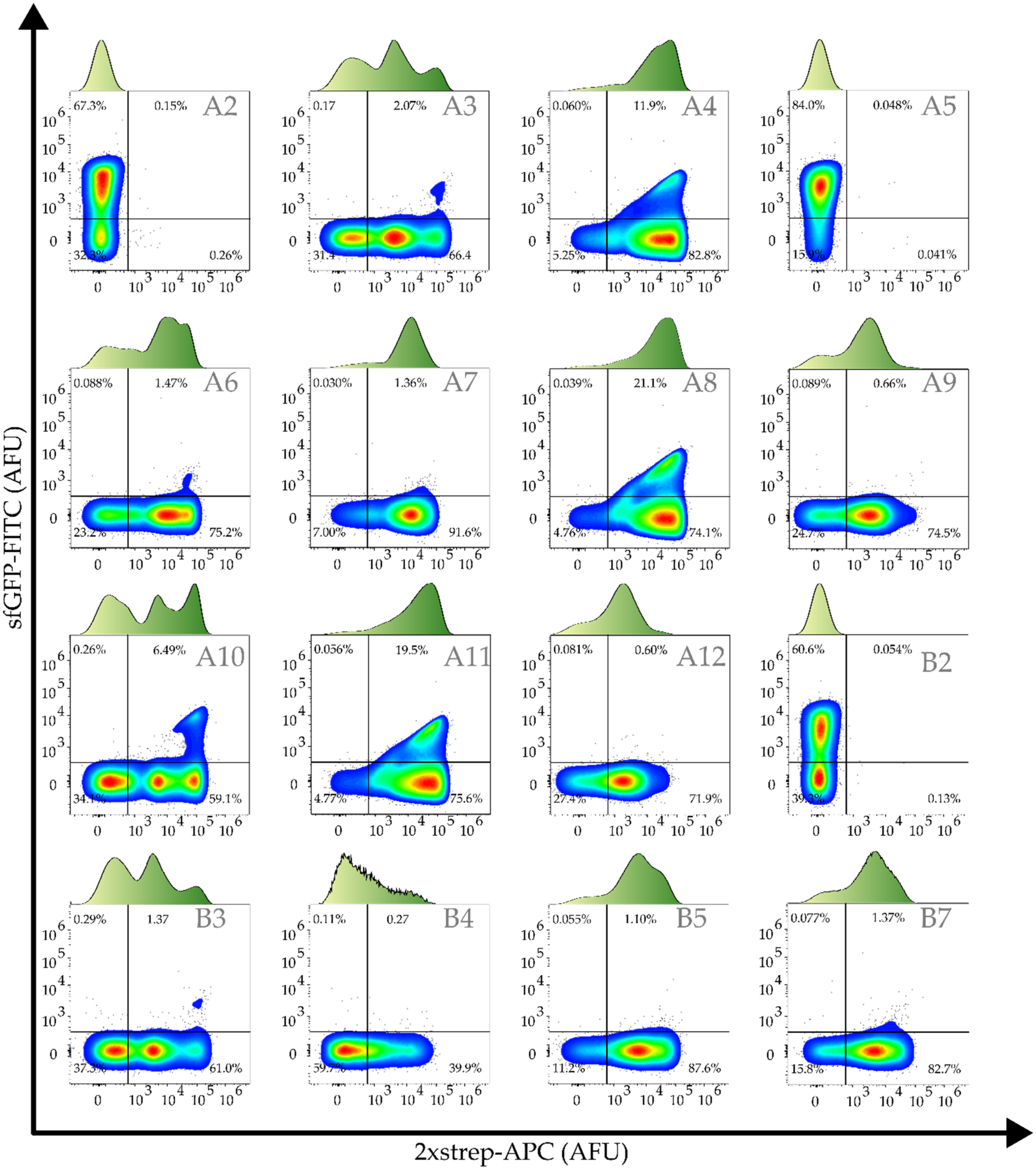

Figure S1. Representative flow cytometric analysis of screened clones (labelled as A2-D12).

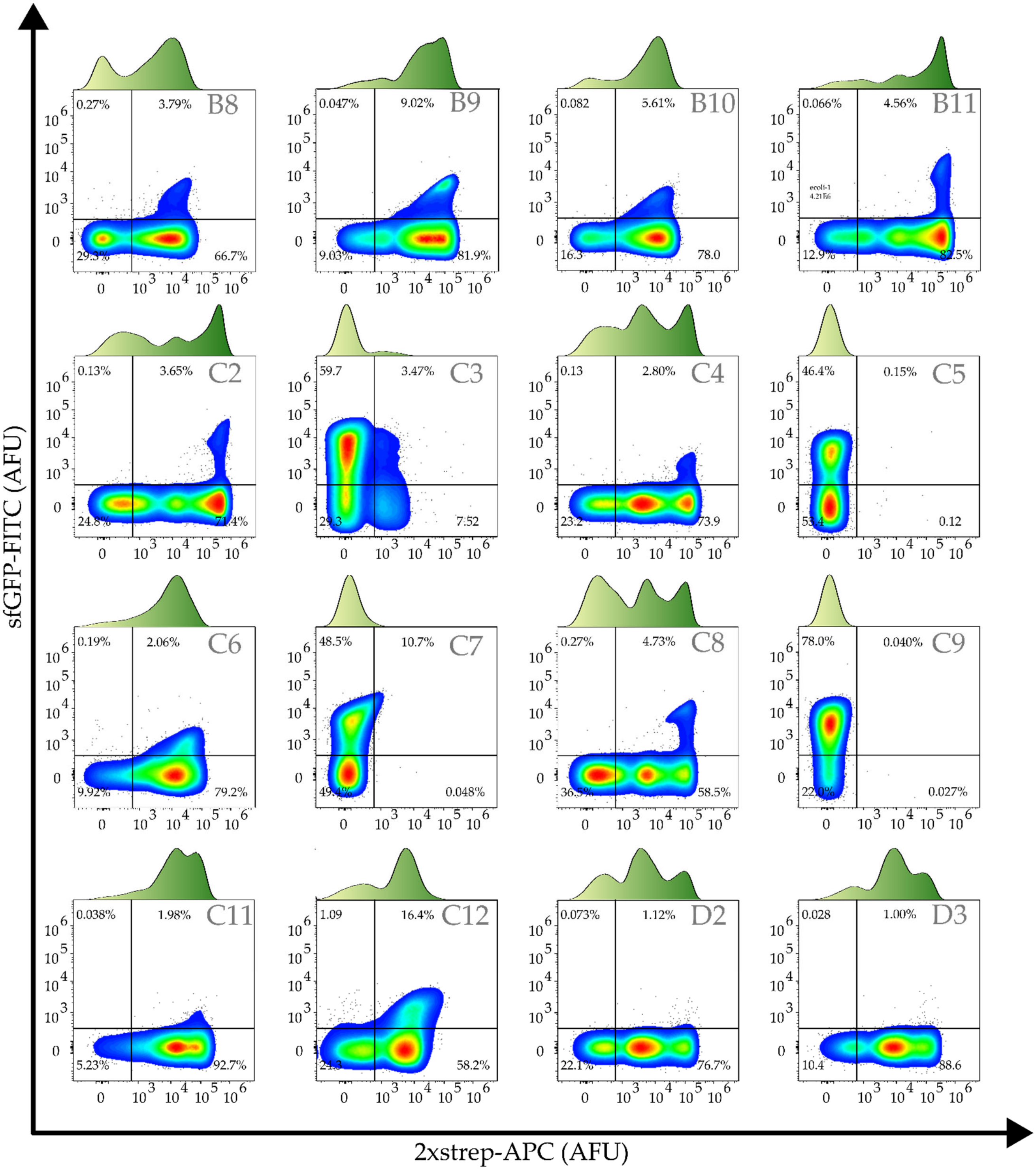

Figure S2. Representative flow cytometric analysis of screened clones (labelled as A2-D12).

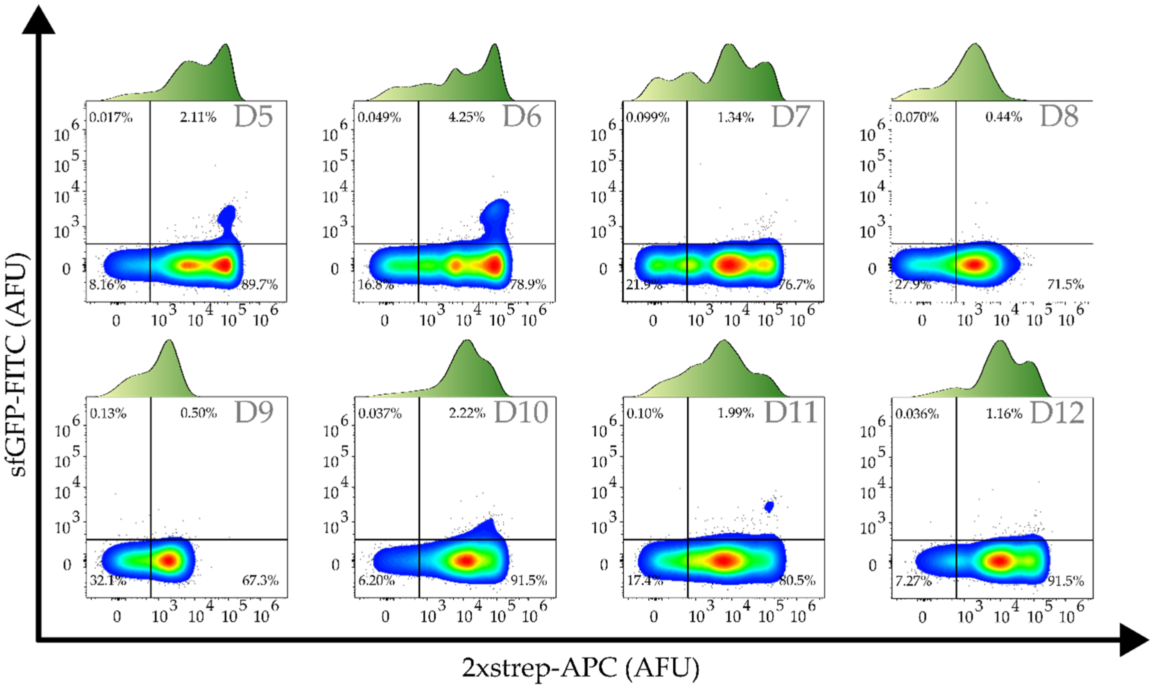

Figure S3. Representative flow cytometric analysis of screened clones (labelled as A2-D12).

(A)

(B)

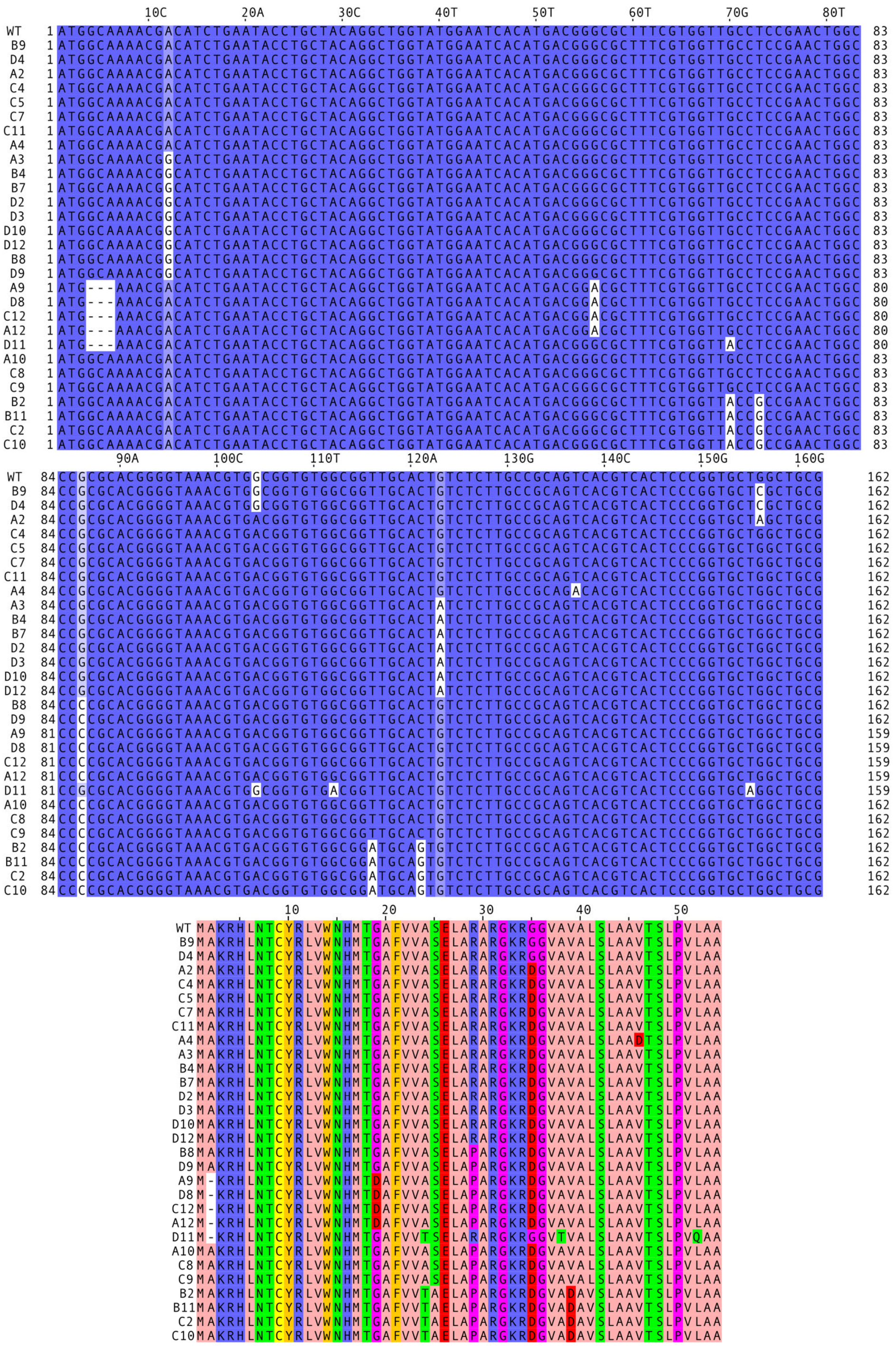

Figure S4. Multiple sequence alignment of the nucleotide and amino acid Ag43 signal peptide sequences of 26 clones. 7 different mutants variants were identified: B9, D4 (156G>C, silent); A2(104G>A, G35D; 156G>A, silent) ; C4, C5, C7, C11(70G>A, A24T; 74T>G, S25A; 86G>C, R29P; 104G>A, G35D; 116T>A, V39D; 121C>G, L41V); A4(104G>A, G35D; 137T>A, V46D); A3, B4, B7, D2, D3, D10, D12 (12A>G, silent; 104G>A, G35D); B8, D9 (12A>G, silent;86G>C, R29P ;104G>A, G35D); A9, D8, C12, A12 (4_7del; 58G>A,G19D; 86G>C, R29P; 104G>A, G35D); D11 (4_7del; 58G>A,G19D;70G>A,A24T; 112G>A; A38T; 115T>A, L52Q); A10, C8, C9 (86G>C, R29P; 104G>A, G35D); B2, B11, C2, C10 (70 G>A, A24T;73T>G S25A; 86G>C, R29P; 104G>A, G35D;116T>A, V39D).
